# Scaling holistic e-health solutions in cancer care using a qualitative realist framework

**DOI:** 10.3389/fpubh.2025.1617857

**Published:** 2025-12-03

**Authors:** Samar J. Melhem, Shereen Nabhani-Gebara, Nailya Bulatova, Ibrahim Alabbadi, Hamzeh Almomani, Rimal Mousa, Ahmed Almousa, Yazan AlRashdan, Omar Nimri, Reem Kayyali

**Affiliations:** 1 Department of Biopharmaceutics and Clinical Pharmacy, School of Pharmacy, University of Jordan, Amman, Jordan; 2 Department of Pharmacy, School of Life Sciences, Pharmacy and Chemistry, Kingston University London, Kingston upon Thames, United Kingdom; 3 Cancer Prevention Department, Director of Cancer Registry, Ministry of Health, Amman, Jordan

**Keywords:** realist evaluation, holistic digital platforms, e-health, cancer care, oncology, middle-income countries, implementation science, digital-health integration

## Abstract

**Background:**

While e-health innovations have advanced cancer-care delivery in high-income countries, middle-income countries (MICs) face distinct systemic, infrastructural, and sociocultural challenges in adopting and scaling digital-health interventions. There is a growing imperative to explore how holistic digital platforms can be implemented effectively in oncology within resource-constrained settings.

**Objective:**

This study aimed to investigate the barriers and facilitators influencing the scalability and adoption of a comprehensive e-health solution in cancer care, drawing on a realist paradigm to determine what works, for whom, and under which circumstances, in the context of Jordan.

**Methods:**

A qualitative study, underpinned by a realist paradigm, was conducted using in-depth, semi-structured interviews with oncology healthcare professionals from diverse clinical settings in Jordan. Interviews were conducted in Arabic, translated into English, and analyzed using a hybrid inductive–deductive framework approach. The Implementation of Change Model guided interpretation of the findings, enabling the identification of multi-level contextual influences.

**Results:**

The analysis yielded a central theme—*Facilitators and Barriers to Implementing a Holistic e-health Solution*—comprising six deductive subthemes derived from the Implementation of Change Model: (A) Innovation; (B) Patients; (C) Healthcare professionals; (D) Organizational context; (E) Social context; and (F) Economic and political context. Across these subthemes, interpretive codes were classified as barriers or facilitators. Participants emphasized the necessity of assessing e-health readiness prior to implementation, and the importance of integrated, cross-sectoral approaches to support scalable, sustainable solutions.

**Conclusion:**

This study provides novel, contextually grounded insights into the implementation dynamics of digital transformation in oncology within MICs. It offers actionable guidance for policymakers and system designers aiming to foster sustainable, equitable digital-health ecosystems.

## 1 Introduction

### 1.1 Global context and evidence gap

The transformative promise of e-health technologies—to enhance care quality, diversify service offerings, extend equitable reach, and contain costs—is now well documented (1–3). Yet most empirical evidence originates from high-income settings, where robust digital infrastructure, comprehensive regulatory frameworks, and a digitally proficient workforce have enabled seamless integration into tertiary-care pathways (4–6). By contrast, health systems in many middle-income countries (MICs) lag behind other information-intensive sectors in their investment in information and communication technologies (ICTs) (6–10). These systems contend with patchy connectivity and regulatory gaps (6), limited patient digital-health literacy (7), uneven digital competence among healthcare professionals (8, 9), and constrained budgets for implementation and maintenance (10). Consequently, harnessing e-health’s full potential in MICs demands parallel investments in technical infrastructure and workforce capacity, co-designed by clinicians and policymakers to secure long-term sustainability (11–13).

### 1.2 From potential to preparedness

Beyond operational efficiencies, digital platforms can foster patient activation and shared decision-making, empowering individuals to participate actively in their own care (13, 14). Nevertheless, rigorous evidence on e-health’s effectiveness for chronic-disease management, including cancer care, in MICs remains sparse. Implementation failures seldom reflect technological deficiencies; rather, they stem from inadequate preparedness at the individual, organizational, and system levels, where interacting barriers multiply risk and squander limited resources (15). As such, systematic appraisal of e-health readiness—encompassing technical capacity, governance structures, and stakeholder attitudes—has emerged as a prerequisite for successful scale-up (14, 15).

### 1.3 Jordanian context and study aim

Jordan offers an instructive case. Recently reclassified by the World Bank from an upper- to a lower-middle-income economy, the country has 10.2 million residents (47% female), with 90.3% living in urban areas and 92% smartphone penetration (16, 17). Health services are delivered through four largely independent sectors: Ministry of Health hospitals, Royal Medical Services, a robust private sector, and semi-government institutions, including university hospitals and the King Hussein Cancer Center (KHCC). However, interoperability across their information systems remains minimal (18, 19). Consequently, even with KHCC’s status as a comprehensive, high-technology oncology center, many patients must traverse multiple providers to complete multimodality care, fracturing clinical data and duplicating diagnostics (18). Previous qualitative work with oncology professionals advocated a national e-health gateway to federate disparate records, embed tele-oncology, and transmit algorithm-guided survivorship plans to trained family physicians; however, technical interoperability alone is insufficient without clear governance, sustainable financing, and workforce capacity (18).

Jordan’s national e-health strategy is led by the Hakeem Program, initiated in 2009 and managed by Electronic Health Solutions (EHS), a non-profit technology company (20). Hakeem aims to create a unified electronic health record (EHR) for every patient across public facilities; implementation, however, has been uneven. While deployment is substantial in Ministry of Health hospitals and primary care centers, adoption in the Royal Medical Services, university hospitals, and the private sector remains limited (21). A 2019 evaluation reported acceptable integration policies and data processing but identified the absence of appropriate middleware—hindering EHR accessibility—and the need for stronger technical support and pharmacy services (22). Coverage gaps persist fewer than 25% of primary-care facilities, and only 25–50% of secondary and tertiary facilities, have implemented an EHR; just 10.3% of hospitals report comprehensive EHR capability (21, 23). In cancer care—predominantly centralized in tertiary centers, with KHCC treating more than 60% of national cases—this fragmentation scatters clinical data across siloed systems, drives duplicative testing, delays treatment, and obscures a coherent longitudinal record; centralization further burdens rural and underserved patients with logistical and financial barriers (18, 23). Low EHR adoption and weak interoperability together impede the development of an integrated, nationwide cancer-care pathway. Against this backdrop, this study examines facilitators and barriers to the nationwide deployment of a supportive digital cancer-care platform in Jordan. By eliciting oncology professionals’ perspectives on e-health readiness and scalability, it generates context-sensitive recommendations to inform policy and practice in Jordan and provides a blueprint for comparable middle-income countries (MICs) seeking equitable cancer outcomes.

### 1.4 Aim

To qualitatively explore oncology professionals’ experiences of, and views on, deploying and scaling up digital health for cancer care in Jordan, elucidating implementation barriers and enablers that can strengthen services.

### 1.5 Objectives

1. Assess facilitators and barriers at the system, organizational and workflow levels for nationwide adoption of digital health in oncology.
2. Characterize multidisciplinary oncology professionals’ perspectives on the use of digital health for supportive cancer services.
3. Identify and prioritize actionable opportunities for implementing digital health initiatives based on stakeholder input.

## 2 Methods

### 2.1 Ethical approval

Ethical approval was granted by Kingston University’s Committee for Scientific Research Ethics (reference 1416).

### 2.2 Study design and recruitment

We conducted a qualitative study using in-depth, semi-structured interviews with oncology healthcare professionals (HCPs). The Participant Information Sheet (PIS; Supplementary file S1) outlining study aims was distributed by email, WhatsApp, or hand delivery. Interviews were held either face to face or via secure videoconference (Skype, Zoom, FaceTime, Google Meet), according to participant preference. Written consent was obtained for in-person interviews; verbal, audio-recorded consent was obtained for virtual interviews. All PIS distributions and consent decisions were logged on a master list.

Sampling proceeded in two sequential phases, each selected to align with the study’s aims and to optimize the relevance and breadth of perspectives. First, purposive sampling (24) was used to identify information-rich participants across public, semi-governmental, and private sectors. This approach was chosen to ensure inclusion of HCPs with direct responsibility for cancer care and practical exposure to digital services; to maximize variation in role, seniority, care setting, and tumor stream (with emphasis on breast and colorectal services); and to achieve analytic depth rather than statistical representativeness. One eligible oncologist declined participation.

Second, snowball sampling (25, 26) was employed to extend recruitment by asking enrolled HCPs to refer peers or share contact details. Snowballing was justified to reach busy or less visible clinicians across dispersed sites, to mitigate gatekeeping barriers, and to broaden the sample efficiently within time and resource constraints while continuing to diversify roles and institutions. The interviewer (SJM) had no prior acquaintance with participants, helping to maintain professional distance and reduce potential selection or response bias. Recruitment continued until thematic saturation was reached.

### 2.3 Data collection

Between July 2022 and December 2023, the first author (SJM) conducted in-depth interviews in Jordanian Arabic with oncology healthcare professionals. All interviews were audio-recorded, transcribed verbatim, and translated into English. To ensure linguistic fidelity, a subset of translations was independently verified by two bilingual co-authors (SNG and RK).

### 2.4 Interview topic guide

The semi-structured interview guide (Supplementary file S2) was developed through an iterative process grounded in a targeted review of the digital-health literature and the study aims (27–30). Candidate questions were drafted to ensure conceptual coverage across 11 domains: personal and professional engagement with digital tools; current practices and attitudes toward recommending online resources; perceived clinical and relational benefits; workflow implications; ethical, legal, privacy, and data-security concerns; integration with patient portals, personal health records (PHRs), and electronic health records (EHRs); system-level enablers and barriers to national scale-up; organizational culture and capacity; patient-level acceptance and equity considerations; professional endorsement and institutional legitimacy; and closing reflections on priorities for policy and research. Items were open-ended with neutral probes to elicit depth, allow unanticipated issues to surface, and minimize leading language.

To enhance face and content validity, the guide was reviewed by clinical and methodological advisors with expertise in oncology, digital/e-health, and qualitative methods. A brief pilot confirmed clarity, thematic flow, and contextual appropriateness; minor wording and ordering refinements were made accordingly. Participants were informed that they could skip any question outside their professional scope.

### 2.5 Data analysis

**Figure 1:**
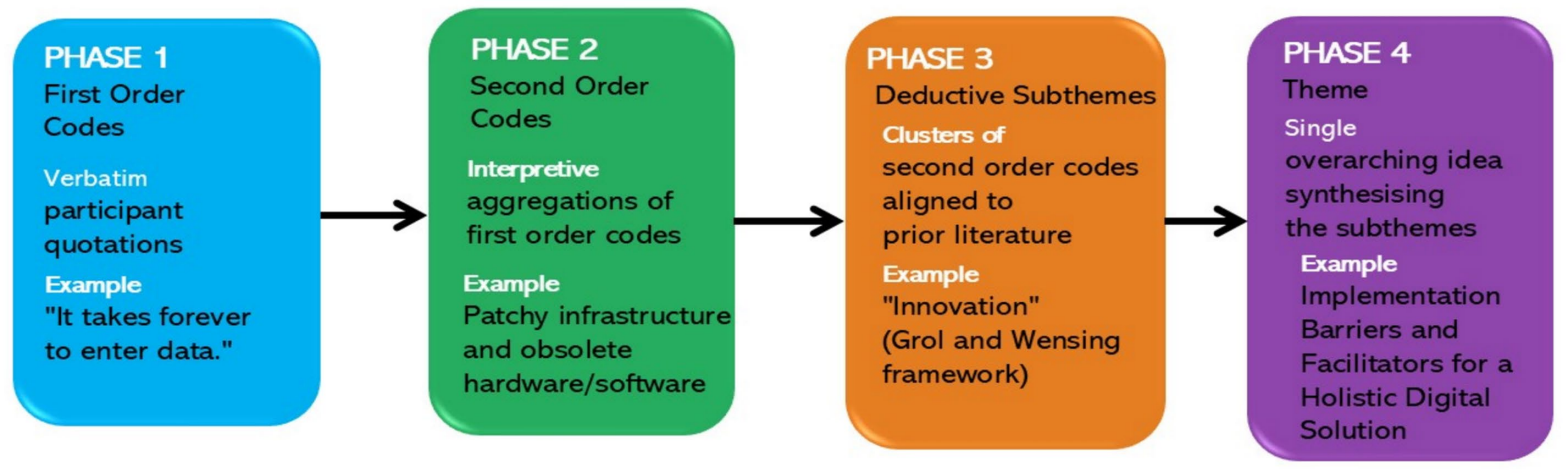
Sequential process of qualitative analysis using the Framework Method. The figure depicts the four-phase analytic workflow, beginning with inductive coding (Phases 1–2) and progressing to deductive alignment with an *a priori* framework (Phases 3–4), following Gale et al.’s hybrid Framework Method.

We employed Gale et al.’s hybrid inductive–deductive Framework Method within a critical-realist epistemology (31, 32). The analysis proceeded through four phases (Figure 1). In Phase 1 (initial coding), the principal investigator (SJM) coded each transcript line by line in NVivo 12 for data management; first-order, data-driven codes were generated inductively from the transcripts and were systematically linked to supporting participant quotations. In Phase 2 (code validation and refinement), RK and SNG reviewed and refined the coding framework to enhance credibility and rigor; related first-order codes were synthesized into second order (interpretive) codes as inductive aggregations grounded in participants’ quotations. In Phase 3 (deductive mapping), interpretive codes were mapped deductively to subthemes derived from the *a priori* concepts of Grol and Wensing’s Implementation of Change Model and Flottorp et al.’s determinants framework (29, 30). In Phase 4 (thematic integration), all subthemes were integrated into a single overarching theme—*Implementation barriers and facilitators for a holistic e-health solution*—which was derived by team consensus, aligned with the primary research question, and reflective of the sociotechnical complexity of embedding a comprehensive e-oncology platform in a middle-income context. Data saturation was reached once no new codes emerged and was verified by three additional interviews, which served only to confirm satisfaction and were excluded from the final analysis (33).

Reporting followed the COREQ criteria (34); the completed checklist is provided in Supplementary file S3. Additional illustrative quotations for each deductive subtheme are presented in Supplementary file S4. Illustrative quotations are presented with the following identifiers: HCP (healthcare professional role), ID (unique participant identifier), specialty, sector, gender, and age.

## 3 Results

### 3.1 Participant characteristics

Twenty-two oncology practitioners representing a range of specialties participated in the study. The mean interview duration was 80 min (range, 53–127 min). As shown in Table 1, the cohort included nine senior consultant oncologists (six men, three women), among them one university hospital director and one senior manager at KHCC; five oncology fellows (four men, one woman); two female clinical nurse specialists; three registered nurses (two men, one woman); and three female board-certified oncology pharmacists. All participants were based in Amman, Jordan.

**Table 1:** Overview of study participants (*N* = 22).

Characteristic	Category	*n* (%)
Age (years)	Range 29–69	
	Median 42	
Sex	Male	12 (54.5)
	Female	10 (45.5)
Role/position	Consultant oncologist (surgical, medical, radiation)	9 (40.9)
	Oncology fellows^†^	5 (22.7)
	Clinical nurse specialist	2 (9.1)
	Registered nurse	3 (13.6)
	Oncology pharmacist	3 (13.6)
Practice setting	Cancer center	5 (22.7)
	University hospital	6 (27.3)
	Public sector hospital	5 (22.7)
	Private sector hospital	4 (18.2)
	Military services	2 (9.1)
Oncology experience (years)	Range^†^ 5–38	
	Median 13	

^†^Fellows (*n* = 5) were internal-medicine graduates undertaking oncology subspecialty training at the cancer center. The field labeled “*Oncology experience (years)*” reports total post-licensure clinical practice to enable comparison across roles; consequently, the minimum observed value is 5 years. Fellows’ oncology-specific exposure at interview was at an early training stage.

### 3.2 Thematic analysis

**Figure 2:**
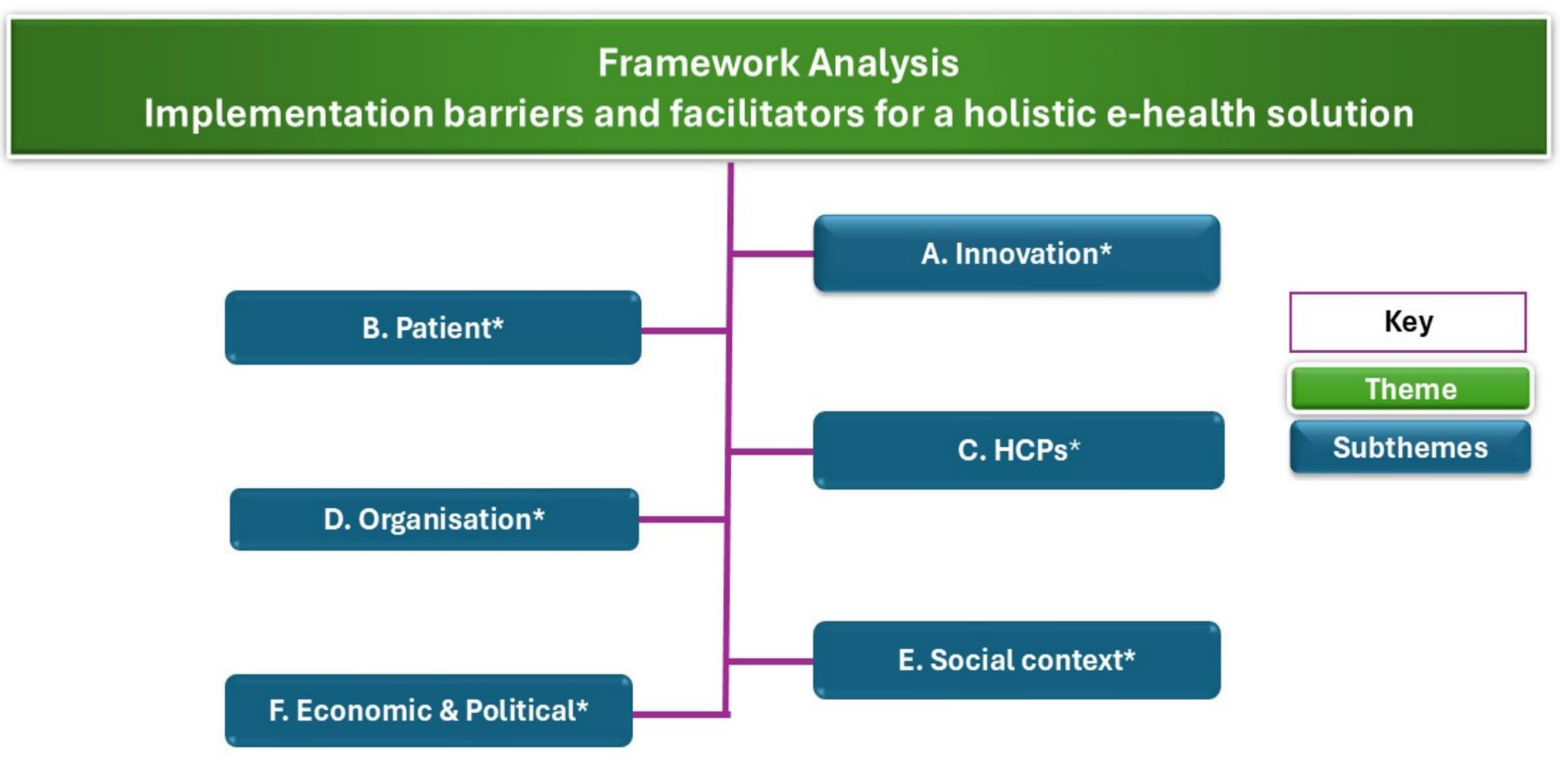
Framework analysis of implementation barriers and facilitators for the scale-up of a holistic e-health solution in a middle-income setting. Framework analysis employed a hybrid inductive–deductive coding approach. All first- and second-order codes were generated inductively. Deductive, *a priori*, subthemes derived from Naderifar et al. (25) and Parker et al. (26) are indicated by an asterisk (*).

The analysis identified one overarching theme, Implementation Barriers and Facilitators for a Holistic e-health Solution, supported by six interrelated subthemes specified *a priori* from implementation frameworks by Naderifar et al. (25) and Parker et al. (26): innovation (A), patients (B), healthcare professionals (C), organizational context (D), social context (E), and economic and political context (F). Within each subtheme, first- and second-order codes were developed inductively and classified as barriers or facilitators. Table 2 summarizes the codes by subtheme, and Figure 2 depicts the relationships among the overarching theme, subthemes, and interpretive codes. The scope of the analysis is supportive care for cancer survivors rather than treatment-specific electronic procedures or disease-specific protocols.

**Table 2:** Implementation barriers and facilitators for a holistic e-health oncology platform.

Deductive subtheme	Inductive second-order codes	
	Barriers	Facilitators
A. Innovation	Patchy infrastructure and obsolete hardware/software Poor interoperability between existing EHRs Low user-friendliness and limited personalization Concerns regarding data security and privacy Uncertain clinical value and limited evidence base Limited hands-on experience among HCPs	Endorsement by respected cancer specialists Not-for-profit, zero-cost model for patients Open-source, standards-based architecture Independent security accreditation and encryption Formal usability and feasibility testing prior to roll-out Third-party management to reduce bureaucratic delay Optional, opt-in use that respects clinical autonomy
B. Patient	Low digital skills, limited education, and low motivation Multiple comorbidities and disease complexity Better fit for chronic disease and survivorship than for active treatment Varying preferences for virtual versus face-to-face care Limited DHL and anxiety regarding results Family gatekeeping and stigma in collectivist contexts	Easier access to HCPs and services (tele-oncology, secure messaging) Greater satisfaction and sense of control Empowerment through personalized education, self-management tools, and survivorship plans Documented information for older adults or patients with cognitive impairment Lifestyle-modification use cases (chronic disease management)
C. Healthcare professionals	Administrative burden from formalized digital communication Understaffing and lack of protected digital time Digital skills and training gaps Perceived threats to professional authority and trust Unclear reimbursement for virtual care	Dedicated digital-care teams and rotation-based virtual clinics (with protected digital hours) Streamlined administrative workflows (electronic scheduling and interoperable electronic documentation) Comprehensive education and training resources Clear remuneration models for teleconsultations and virtual care
D. Organizational context	Weak leadership commitment and IT-investment priorities Hierarchical bureaucracy and resistance to change High up-front costs and broad project scope (lack of piloting) Patchy digital culture across hospitals	New governance unit with authority, budget, and staff Intra- and inter-organizational interoperability targets (national patient-access system) Pilot projects and staged scale-up linked to service-quality metrics
E. Social context	Collectivist norms influencing information flow “Wasta” (connections/influence) and informal pathways undermining transparency Digital divide affecting rural, older, and disabled groups	Multilevel public-engagement campaigns Leveraging high urban smartphone penetration Community health-worker outreach and tailored initiatives
F. Economic and political	Slow regulatory processes and lack of a unified national e-health strategy Ambiguity regarding liability and privacy legislation Limited public funds and payer reluctance Low entrepreneurial appetite and fragmented initiatives	Strategic planning and sustainable financing Low-cost reforms framed as efficiency gains Crisis-response experience (COVID-19 and regional emergencies) leveraged as investment catalysts

EHRs, electronic health records; HCPs, healthcare professionals; IT, information technology; DHL, digital health literacy; HL, health literacy.

“Wasta” is an Arabic term that loosely translates to “connections,” “clout,” or “influence.” It refers to a pervasive socio-cultural practice in Middle Eastern societies where social networks and influence are used to obtain services or opportunities. While often perceived as a form of nepotism or corruption, it is also regarded as a pragmatic mechanism to navigate bureaucratic, social, or professional barriers in Jordanian society (35, 36).

Operational definitions are as follows. Innovation denotes attributes of the e-health solution that shape adoption, including perceived clinical value, usability, interoperability, workflow fit, evidence base, and security. The patient’s domain encompasses capabilities and preferences, caregiving dynamics, and access to devices and connectivity. The healthcare professional’s domain covers skills and training, attitudes toward digital care, workload and role clarity, incentives, and perceived liability. Organizational context comprises leadership and governance, culture and readiness for change, staffing and protected time, information-technology infrastructure, and financing. Social context refers to prevailing norms and informal networks (for example, family mediation and wasta), language and communication expectations, and urban–rural disparities. The economic and political context includes policy and regulation, reimbursement and payment models, market structure, and fiscal constraints. This deductive scaffold (Table 2; Figure 2) structured coding and comparison, while inductive development of interpretive codes preserved context-specific insights from participants.

## 4 Results

### 4.1 Innovation-level factors

Several obstacles hinder the successful implementation of new e-health technologies. Technical shortcomings, for instance, such as outdated hardware and insufficient bandwidth, create significant barriers. The poor interoperability between different electronic health record (EHR) systems is another major issue, which often necessitates a continued reliance on paper-based documentation and can lead to the duplication of clinical investigations.

These integration challenges demand substantial investments in technical infrastructure, further complicating the deployment of new systems. However, an effectively designed e-health platform has the potential to address these problems. By functioning as a shared longitudinal record and an integration layer, it could securely store essential patient data and enable controlled data flows across various hospital systems, even in the absence of full cross-sector interoperability. As one participant noted, *“Some places aren’t fully computerized yet, so they still have to do paperwork…” (HCP12, oncology pharmacist, private sector, female, 36 years).*

Concerns regarding data security and the capacity to manage large volumes of sensitive information also present considerable challenges. One oncology pharmacist expressed her reservations, stating, *“Most critically, the new platform must provide data encryption and security and manage its massive data output. Integrating existing electronic health record systems may be difficult; they need servers. I’m not convinced about IT preparedness.”* (HCP13, oncology pharmacist, private sector, female, 33 years).

Furthermore, respondents highlighted how inefficient referral processes and incompatible patient identifiers contribute to an increased administrative burden and delays in care. A clinical nurse specialist explained, *“…referrals stall because identifiers from other sectors, such as the private sector, are not recognized. We fax protocols, then type them in again, resulting in duplication of effort and avoidable delays for patients.” (HCP15, clinical nurse specialist, KHCC, semi-governmental, female, 37 years).*

Beyond the technical and administrative hurdles, the adoption of new e-health platforms is also hindered by issues of usability and clinical relevance. Several professionals emphasized that low usability and limited personalization of existing platforms are significant barriers to adoption, and they stressed the critical importance of user-centered design. A senior fellow oncologist suggested, *“The app should provide simple and stage-specific general information about the disease, be personalized for each case, and have an interface that is flexible and accommodating for data entry. Ultimately, a user-friendly technology that streamlines healthcare professionals’ workflows would improve patient care.” (HCP10, senior fellow oncologist, KHCC, semi-governmental, male, 40 years).*

Other clinicians questioned the clinical relevance and evidence base of some digital tools, arguing that robust proof of their impact on patient outcomes is necessary before endorsing investment. As one surgical oncologist put it, *“Spending on platforms whose impact on patient outcomes remains uncertain is a frivolous luxury… They may help improve service delivery…for patients’ outcomes…pretty unsure.” (HCP8, surgical oncologist, KHCC, semi-governmental, male, 52 years).*

Finally, a limited prior exposure to e-health platforms has fostered a sense of reluctance among some healthcare professionals, highlighting the need for comprehensive training and a phased roll-out of new technologies. *“Though the idea of a holistic platform you are talking about may sound appealing… I never saw that.” (HCP14, clinical nurse specialist, KHCC, semi-governmental, female, 34 years).*

On the other hand, several factors were identified that could facilitate the adoption of e-health technologies. The endorsement of respected specialists and a transparent affiliation with reputable institutions, such as the Ministry of Health, can lend legitimacy to a new platform and promote its uptake. One participant stated, *“Yes, we need prominent cancer doctors to support the app, and it should have transparent affiliations with institutions or the Ministry of Health to increase user adoption.” (HCP12, oncology pharmacist, private sector, female, 36 years).* The availability of non-profit, freely accessible solutions can also help to lower cost barriers and broaden access to these technologies. As a medical oncologist noted, *“Most of them [patients] have insurance, so I doubt they’ll pay for such a service, especially when you start it…” (HCP7, medical oncologist, public sector, male, 49 years).* Moreover, the use of open-source platforms that enable interoperability across different institutions may enhance data exchange while still preserving the necessary access controls. *“The system should be open source for interoperability with other providers, while remaining proprietary to guarantee that only eligible patients access our services.” (HCP2, senior oncology manager, KHCC, semi-governmental, female, 56 years).*

In addition to these factors, the establishment of robust, regulatory-compliant data-privacy safeguards is essential for engendering trust among users. As one senior oncology manager emphasized, *“The exchange, usage, and storage of data are crucial in patient-related matters, necessitating established protocols.” (HCP2, senior oncology manager, KHCC, semi-governmental, female, 56 years).*

The implementation of pilot programs with rigorous usability testing and third-party management can also help to strengthen technical reliability and support for new platforms. A surgical oncologist suggested, *“While centralizing planning and collaborative scope definition are key, decentralizing implementation allows hospitals to circumvent government bureaucracy through third-party software management.” (HCP1, surgical oncologist, university hospital director, semi-governmental, male, 69 years).* Finally, respondents favored a gradual roll-out of new technologies, with limited-access trials to assess demand, safety, and viability before a full-scale implementation. *“Initially, virtual services access should be restricted to a specific patient group, allowing for evaluation of interest from both patients and providers prior to expanding the program.” (HCP1, surgical oncologist, university hospital director, semi-governmental, male, 69 years).*

### 4.2 Patient-level factors

The adoption of e-health technologies by patients is influenced by a variety of individual factors. Limited digital literacy, lower levels of educational attainment, and varying degrees of motivation can all reduce a patient’s ability to engage effectively with digital tools. As one medical oncologist observed, *“…I envisage most likely those well-educated, tech-savvy, predominantly good-prognosis patients…” (HCP10, medical oncologist, private sector, female, 57 years).* Clinical characteristics, such as the presence of comorbidities, a patient’s tendency to seek out information, and the stage of their cancer, also play a role in uptake. Thoughtful design that aligns with patient capabilities may help to mitigate some of these barriers.

Clinicians also noted that digital applications are often better suited to survivorship and chronic care than to patients who are undergoing active treatment or experiencing significant psychological distress. An oncology pharmacist shared*, “Patients are expected to be compliant by recording their symptoms and feelings; however, they often become bored. As you know, even with medications, we still face issues of non-adherence.” (HCP11, oncology pharmacist, private sector, female, 37 years).* Furthermore, a preference for in-person care can shape uptake, particularly when it comes to emotionally charged or complex decision-making. *“…I do not think the app can handle intricate conversations about challenging decisions, especially if the findings are crucial or require follow-up…” (HCP3, medical oncologist, private sector, female, 57 years).*

Limited health and digital-health literacy can also generate uncertainty about the outputs of algorithms and reinforces the need for clinician oversight. A surgical oncologist commented, *“We have many AI tools now, but they can be dangerous and cause patient anxiety since we do not know how the algorithms work… There should always be a practitioner monitoring the situation, particularly in the early stages after diagnosis.” (HCP8, surgical oncologist, KHCC, semi-governmental, male, 52 years).*

In more collectivist cultural contexts, family decision-making can either facilitate or impede the uptake of e-health technologies, especially in situations where families control the disclosure of a diagnosis. *“…when the family decides not to notify the patient about his diagnosis, we often observe that the patient is old and the family is scared… this is intensely uncomfortable for me as the family can be angry and violent.” (HCP2, senior oncology manager, KHCC, semi-governmental, female, 56 years).*

There are, however, a number of factors that can facilitate patient adoption of e-health technologies. Telemedicine and secure messaging, for example, can strengthen the patient-provider relationship by improving communication and making clinicians more accessible. Some participants argued that building a comprehensive telemedicine infrastructure would be more valuable than developing isolated applications. *“As a doctor, I do not see much point in supportive cancer apps. They might be useful for patient education and supportive care, but I’d rather start and focus on telemedicine program development and implementation.” (HCP1, surgical oncologist, university hospital director, semi-governmental, male, 69 years).*

Indeed, survivors of breast and colorectal cancer have reported greater satisfaction with their care when digital tools were used to streamline access and improve communication. *“If patients believe their clinicians are more accessible because of these tools, I believe satisfaction with care and the quality of healthcare as a whole will improve.” (HCP10, senior clinical fellow, KHCC, semi-governmental, male, 40 years).*

Personalized education, self-management resources, and tailored survivorship plans can also empower patients and lead to better outcomes. Clinicians noted that these tools can be particularly beneficial for older adults and patients with cognitive impairments who may have difficulty remembering verbal instructions. *“Patients with cognitive decline often forget what I tell them and ask again when they get home. This app could be used as a backup to document and distribute information to these patients.” (HCP3, medical oncologist, private sector, female, 57 years).*

It was also suggested that these platforms would be useful for managing chronic conditions that require lifestyle modifications and continuous monitoring. *“…these benefits can be extended to patients with other chronic diseases, especially diabetes and hypertension… and other public health initiatives…” (HCP3, medical oncologist, private sector, female, 57 years).*

### 4.3 Healthcare professional-level factors

The formalization of clinical documentation for patient access can increase the administrative burden on healthcare professionals if it is not supported by clear workflows and a clear delineation of roles. One senior fellow oncologist noted, *“Granting patients access to clinical notes is problematic… Simplifying these notes for patients would be beneficial, but adding this task to healthcare professionals’ workload is impractical.” (HCP10, senior fellow oncologist, KHCC, semi-governmental, male, 40 years).*

Understaffing and the lack of protected “digital hours” for healthcare professionals also constrain the adoption of new technologies. This has led to calls for the creation of dedicated digital service teams rather than assigning these responsibilities on an *ad hoc* basis. *“Hospitals face a critical shortage of skilled clinical nurse specialists… Enhancing nurse training and system use is essential for delivering greater value.” (HCP15, clinical nurse specialist, KHCC, semi-governmental, female, 37 years).*

Some clinicians also expressed a fear that patient reliance on external sources of information could undermine their professional authority and disrupt their workflows. *“Some doctors are resistant to patients consulting external sources, feeling it undermines their authority and disrupts their work.” (HCP11, oncology pharmacist, private sector, female, 37 years).* Furthermore, the lack of clear reimbursement arrangements for virtual care reduces the willingness of healthcare professionals to participate in these initiatives and jeopardizes their long-term sustainability. *“Establishing clear remuneration models for teleconsultations and virtual care was cited as essential to incentivize healthcare professionals’ participation…” (HCP13, oncology pharmacist, private sector, female, 33 years).*

To address these challenges, participants recommended the establishment of dedicated digital-care teams, rotation-based clinics, and protected digital hours to support the delivery of virtual services. *“To carry out this program, you should establish digital care teams.” (HCP2, senior oncology manager, KHCC, semi-governmental, female, 56 years).*

Streamlined administrative processes, electronic scheduling, and interoperable documentation were also expected to free up clinician time for direct patient care. *“The software should be developed to add synergy to my work and help me undertake my responsibilities more efficiently…” (HCP22, fellow oncologist, university hospital, semi-governmental, male, 31 years).*

The integration of comprehensive education resources into care pathways can also improve psychosocial support, decision-making, and patient adherence to treatment plans. *“Educational apps or appointment scheduling tools will not harm provider–patient relationships; they could enhance communication if they offer robust educational support.” (HCP2, senior oncology manager, KHCC, semi-governmental, female, 56 years).*

Finally, clear compensation for teleconsultations and other forms of virtual care was viewed as essential to motivate the participation of healthcare professionals. *“…either the effort needs to be part of our jobs, or we need to receive additional pay… I believe it is unrealistic and impractical to include health practitioners in the app without compensating them…” (HCP15, clinical nurse specialist, KHCC, semi-governmental, female, 37 years).*

### 4.4 Organizational-level factors

Leadership commitment is a pivotal factor in the successful adoption of digital innovation. Without executive buy-in, resource allocation and the strategic prioritization of digital projects can falter. Hierarchical structures and procedural formalities can further complicate the adoption of innovation. As one clinical nurse specialist stated, “For the platform’s success, leadership buy-in and top-down implementation are vital, as are training and reimbursement for health teams…” (HCP15, clinical nurse specialist, KHCC, semi-governmental, female, 37 years).

Competing priorities and constrained budgets often lead to digital projects being relegated below more urgent clinical imperatives, which can result in underinvestment in infrastructure, staff training, and ongoing maintenance. A senior oncology manager explained, *“We have a portfolio of projects to evaluate… we must also consider how each project… can contribute to resolving problems in service delivery in terms of cost-effectiveness and impact on performance.” (Senior oncology manager, KHCC, semi-governmental, female, 56 years).*

Corporate cultures that are resistant to change also pose a significant risk, as there is a tendency to revert to familiar practices once the champions of a new initiative depart. *“If change is implemented in a top-down manner, the status quo will likely return once a top-level manager leaves the organization.” (HCP8, surgical oncologist, KHCC, semi-governmental, male, 52 years).* The high up-front costs and inadequate project scoping, especially when not preceded by a pilot phase, can also render comprehensive platforms unwieldy and difficult to manage. *“The devil is in the details, so if we are talking about a comprehensive digital platform for cancer patients, that’s a big project.” (HCP14, clinical nurse specialist, KHCC, semi-governmental, female, 34 years).*

To overcome these barriers, it is recommended to create dedicated digital-care departments staffed by trained specialists with delegated authority and defined budgets. This can provide the necessary leadership and reduce workflow disruption. *“To reduce workflow disruption, I suggest creating a dedicated department for these services…” (HCP7, medical oncologist, public sector, male, 49 years).*

Inter- and intra-organizational collaboration, combined with explicit interoperability targets and a national patient-access system, can also help to prevent fragmentation and enable the secure exchange of data. “*Collaboration is essential to develop a national patient access system that securely connects our facility with most others…” (HCP1, surgical oncologist, university hospital director, semi-governmental, male, 69 years).*

Finally, the use of pilot projects, a staged scale-up, and clear service-quality metrics (e.g., usability and outcome improvement) were preferred to demonstrate the value of a new platform prior to its broad expansion. *“If the platform is not effective or does not enhance services, users will find alternative channels… Success hinges on reducing costs and elevating service quality.” (HCP9, oncology radiologist, public sector, female, 46 years).*

### 4.5 Social-context-level factors

Multi-level public engagement that involves clinicians, policymakers, and technology firms can foster more positive attitudes toward e-health and accelerate its adoption, particularly in urban areas with high levels of connectivity. One fellow from a university hospital noted, *“With effective promotion across multiple levels, especially in large cities… the use of these tools is likely to increase significantly.” (HCP21, fellow, university hospital, semi-governmental, male, 31 years).*

The high penetration of smartphones in urban areas also offers the potential for a rapid scale-up of e-health platforms, provided they are built on robust architecture and institutionalized within existing care pathways, rather than being launched as stand-alone commercial applications. *“Nearly everyone has one or two smartphones, which can enhance reach; however, success ultimately hinges on the solution’s architecture and its institutionalization within care pathways…” (HCP20, fellow, university hospital, semi-governmental, female, 30 years).* Community health-worker outreach and other tailored initiatives can also help to reduce digital disparities by addressing gaps in familiarity with technology and other access barriers. “*Many people may be resistant to the idea of virtual doctor visits because they lack familiarity with the technology involved… It will be especially difficult to convey the message to patients in disadvantaged settings such as refugee camps.” (HCP15, clinical nurse specialist, KHCC, semi-governmental, female, 37 years).*

### 4.6 Economic and political context

Slow regulatory processes can impede progress in the adoption of e-health and create uncertainty about how digital initiatives should be structured and governed. A surgical oncologist and university hospital director commented, *“Regulatory processes slow us down. For digital initiatives to be sustainable, it’s crucial to involve multiple sectors…” (HCP1, surgical oncologist, university hospital director, semi-governmental, male, 69 years).*

The absence of a unified national e-health strategy also limits the potential for coordinated transformation and the setting of clear goals across the health system. *“At present, there is no overarching national digital health strategy to direct the healthcare sector’s transformation.” (HCP1, surgical oncologist, university hospital director, semi-governmental, male, 69 years).* Ambiguities surrounding liability and privacy legislation can fuel concerns about the commercialization of healthcare and the potential risks for clinicians. “There’s a worry that unchecked, profit-motivated development of digital health apps could lead to more chaos and disruption in healthcare…” (HCP3, medical oncologist, private sector, female, 57 years).

Limited public funds and a reluctance on the part of payers can also constrain the development of sustainable financing models and delay the scale-up of e-health initiatives.

*“…to convince payers and other stakeholders, who can be a major stumbling block to the implementation and scalability of digital care initiatives…” (HCP1, surgical oncologist, university hospital director, semi-governmental, male, 69 years).*

On the other hand, strategic planning and predictable financing can catalyze the adoption of e-health by aligning stakeholders, clarifying priorities, and ensuring a stable source of resourcing. *“As technology becomes more affordable, it offers potential improvements. However, success requires collective effort. The main challenge lies in planning, execution, and financing…” (HCP4, hematologist-oncologist, military services, male, 47 years).*

Low-cost reforms that are framed as efficiency gains can also help to advance implementation when budgets are constrained, particularly when the proposed solutions can demonstrably reduce waste or administrative burden. Finally, the experience of crisis-response, for example, the use of telemedicine during emergencies, can accelerate coordination and create momentum for the institutionalization of e-health technologies. “*Telemedicine is essential in times of crisis; the armed forces make use of it on an as-needed basis.” (HCP6, surgical oncologist, military services, male, 63 years).*

## 5 Discussion

Adopting a realist perspective, this study indicates that the viability of a holistic digital oncology platform in a middle-income context depends less on technological sophistication than on the coherent alignment of six interdependent domains: innovation, patients, healthcare professionals, organizational context, social context, and economic and political context. By elucidating context-specific mechanisms—such as collectivist family mediation, clinicians’ workload calculus, and the informal practice of *wasta*—our findings extend an evidence base that is largely grounded in high-income settings and offer a contextually attuned, transferable framework for middle-income countries seeking to design scalable, equitable e-health solutions. We discuss findings across six *a priori* subthemes: innovation (A), patients (B), healthcare professionals (C), organizational context (D), social context (E), and economic and political context (F) and use these labels consistently to mirror Table 2. The scope pertains to supportive care for cancer survivors rather than treatment-specific electronic procedures or disease-specific protocols.

At the patient level, uptake reflected the interplay between individual capability and communal norms. Low health and digital-health literacy limited patients’ ability to self-navigate, yet family members frequently compensated for technical deficits, reflecting collectivist expectations and the influence of *wasta* in institutional interactions (35, 36). Designing platforms that address both patients and caregivers, use vernacular Arabic, and embed culturally resonant messaging can convert exclusionary mechanisms into inclusive ones, thereby enhancing equity of access.

Professional engagement proved equally pivotal. Clinicians expressed concerns about workload, medico-legal exposure, and erosion of empathic contact (37, 38); however, these concerns diminished when tele-oncology hours, remuneration, and documentation standards were formalized. Linking license renewal to demonstrated digital competency (39) and reimbursing virtual consultations at parity with face-to-face care can reframe e-health as an efficiency gain rather than an added administrative burden, thereby activating professional ownership. Technology architecture conditioned trust: open-source, bilingual, low-bandwidth-tolerant platforms that integrate with existing systems foster confidence, consistent with recent reviews (40), whereas proprietary “black-box” solutions generate skepticism (41, 42). Staged pilots with publicly reported dashboards, together with procurement policies that prioritize modular, standards-based designs, are therefore essential for successful scale-up.

Even robust technology falters without organizational readiness. Fragmented information systems and ambivalent leadership inhibited routinization (43, 44). Where executives invested in e-oncology teams and set explicit digital-maturity targets, collective learning and adoption accelerated (45–47). Tying hospital accreditation and public oncology contracts to demonstrable digital capability would create strong incentives for sustained investment. These findings do not presuppose fully standardized hospital structures; they were observed across public, private, university, and military settings with variable pathway maturity. Where tumor-specific pathways (for example, breast units) are more standardized, the same mechanisms as leadership, protected digital time, interoperability, and remuneration tend to accelerate routinization rather than change its direction.

Culture and politics add further complexity. Collectivist decision-making can delay disclosure of diagnoses, and *wasta* can distort referral pathways (35, 36). Platforms embedded with transparent triage rules and audit trails can mitigate such distortions, but they must also address the digital divide; without subsidized data bundles and community access points, reforms risk amplifying inequities rather than narrowing them. Finally, the absence of comprehensive e-health legislation leaves liability and data stewardship unclear, mirroring patterns observed across many low- and middle-income countries (LMICs) (48). A national e-health statute that codifies privacy protections, cross-border licensing, and equitable reimbursement would provide an enabling environment for sustainable diffusion; alignment with international standards could further support regional collaboration and subsidize domestic care.

## 6 Strengths and limitations

This study has several strengths. The purposive, multiprofessional sample—comprising oncologists, specialist nurses, and oncology pharmacists—offers a richly triangulated account of e-health readiness that is uncommon in the Middle East. Applying a realist analytic framework illuminated how context–mechanism configurations operate across patient, provider, organizational, and macro-policy strata, generating insights that are theoretically robust and practically transferable to other middle-income settings with collectivist cultures, constrained infrastructure, and emerging tele-oncology agendas.

Several limitations temper interpretation. First, the sample predominantly included clinicians who manage breast and colorectal cancers; consequently, the perceived needs and e-health solutions identified may not fully represent those relevant to other tumor groups. Second, although participants were drawn from public, private, and military sectors, most were urban based, potentially under-representing rural digital realities and bandwidth constraints. Third, this inquiry captured professional perspectives only; patients and caregivers were not interviewed directly, so inferences about end-user experience remain indirect. Finally, as with all interview studies, findings may be influenced by social desirability bias and researcher positionality, despite reflexive measures taken during data collection and analysis. Future mixed-methods and longitudinal research that incorporates patient voices and rural sites would strengthen external validity and track how identified mechanisms unfold during scale-up.

## 7 Conclusion

This study provides a nuanced, system-aware analysis of the conditions required to embed a holistic e-health platform within oncology care in middle-income countries. Using realist lens, we clarify the complex relationships among individual agency, institutional structures, and policy environments that shape digital-innovation adoption. Success depends not solely on technological capability but on aligning multiple enabling conditions: digitally literate patients and caregivers who feel empowered rather than marginalized; clinicians who regard digital care as integral rather than peripheral; interoperable systems that reduce friction rather than add burden; and governance mechanisms that secure trust through ethical rigor, cultural sensitivity, and policy coherence.

Implementation is not a linear checklist but a negotiated, co-constructed process requiring sustained investment, stakeholder dialog, and iterative adaptation. Within this negotiated space, e-health platforms can evolve from fragmented pilots into meaningful, scalable solutions embedded in everyday oncology practice. In contexts such as Jordan—reflective of broader middle-income-country challenges—effective design must leverage local capacities, anticipate socio-political tensions, and build legitimacy among professionals and the public. Ultimately, digital transformation in cancer care entails reconfiguring relationships among people, data, institutions, and care pathways so that e-health becomes structural, equitable, and sustainable.

## 8 Practical and policy implications

To operationalize these findings, national cancer-control strategies in middle-income countries should prioritize digital readiness at both patient and provider levels. This includes embedding digital-literacy interventions across the oncology pathway to ensure that patients and caregivers can confidently engage with patient portals, remote-monitoring systems, and tele-oncology services. Regulators should require demonstrable digital competencies as part of ongoing clinical accreditation and ensure that virtual consultations are reimbursed on a par with in-person visits to legitimize and incentivize adoption.

From a systems perspective, the future of digital cancer care in middle-income countries lies in developing home-grown, open-source platforms tailored to local needs and existing infrastructure. In an era of constrained public and donor funding, governments should commit to procuring open, standards-based digital architectures that promote interoperability, enable incremental upgrades, and avert costly vendor lock-in (42, 48). Public-financing instruments can accelerate this shift by linking oncology investments and service contracts to measurable digital-maturity indicators—such as secure data exchange, clinician uptake, and patient-portal usage.

Finally, enacting a comprehensive e-health statute is imperative to enshrine principles of equity, privacy, and accountability, thereby bolstering public trust and preventing digital exclusion. Future research should employ hybrid effectiveness–implementation designs to trial policy mechanisms and anticipate governance complexities arising from AI-driven oncology applications. Only through deliberate, context-aware, and inclusive strategies can digital innovation achieve its transformative potential in middle-income-country cancer care.

## Data Availability

The original contributions presented in the study are included in the article/Supplementary material, further inquiries can be directed to the corresponding author.

## References

[1] World Health Organization. Atlas of eHealth country profiles: the use of eHealth in support of universal health coverage. Based on the findings of the third global survey on eHealth 2015 [internet]. Geneva: WHO; (2016). Available online at: https://apps.who.int/iris/handle/10665/252529 (Accessed April 10, 2025).

[2] World Health Organization. WHO compendium of innovative health technologies for low-resource settings, 2011–2014: assi*s*tive devices, eHealth solutions, medical devices, other technologies, technologies for outbreaks [internet]. Geneva: WHO; (2015). Available online at: https://apps.who.int/iris/handle/10665/204530 (Accessed April 10, 2025).

[3] Alkmim MB Figueira RM Marcolino MS Cardoso CS Abreu MP Cunha LR . Improving patient access to specialised health care: the telehealth network of Minas Gerais, Brazil. Bull World Health Organ. (2012) 90:373–8. doi: 10.2471/BLT.11.099408 22589571 PMC3341691

[4] Soloe C Burrus O Subramanian S. The effectiveness of mHealth and eHealth tools in improving provider knowledge, confidence, and behaviours related to cancer detection, treatment and survivorship care: a systematic review. J Cancer Educ. (2021) 36:1134–46. doi: 10.1007/s13187-021-01961-z 33598832 PMC7889413

[5] Armaou M Araviaki E Musikanski L. Ehealth and mHealth interventions for ethnic minority and historically underserved populations in developed countries: an umbrella review. Int J Community Well-Being. (2020) 3:193–221. doi: 10.1007/s42413-019-00055-5

[6] World Health Organization. Global diffusion of eHealth: making universal health coverage achievable. Report of the third global survey on eHealth [internet]. Geneva: WHO; (2017). Available online at: https://apps.who.int/iris/handle/10665/252529 (Accessed April 10, 2025).

[7] Melhem SJ Nabhani-Gebara S Kayyali R. Digital trends, digital literacy, and e-health engagement predictors of breast and colorectal cancer survivors: a population-based cross-sectional survey. Int J Environ Res Public Health. (2023) 20:1472. doi: 10.3390/ijerph20021472, PMID: 36674237 PMC9860554

[8] Ruxwana N Herselman M Pottas D. A generic quality assurance model (GQAM) for successful e-health implementation in rural hospitals in South Africa. Health Inf Manag J. (2014) 43:26–36. doi: 10.1177/183335831404300104, PMID: 27010686

[9] Aminu M Phillips E Kolanko C. The role of digital competence in CME uptake: a short communication. J Eur CME. (2022) 11:2019436. doi: 10.1080/21614083.2021.2019436, PMID: 34992950 PMC8725721

[10] Babatunde AO Abdulazeez AO Adeyemo EA Uche-Orji CI Saliyu AA. Telemedicine in low- and middle-income countries: closing or widening the health inequalities gap. Eur J Environ Public Health. (2021) 5:em0075. doi: 10.21601/ejeph/11021

[11] Shuvo TA Islam R Hossain S Evans JL Khatun F Ahmed T . Ehealth innovations in LMICs of Africa and Asia: a literature review exploring factors affecting implementation, scale-up and sustainability. Health Care (Amst). (2015) 2015:95–106. doi: 10.2147/IEH.S88809

[12] World Health Organization. Digital health [internet]. Geneva: WHO; (2025). Available online at: https://www.who.int/health-topics/digital-health (Accessed April 10, 2025).

[13] Binsar F Kartono R Bandur A Kosasih W. Digital transformation of information fulfilment and patient engagement for health-service safety. In: Proceedings of the 4th international conference on management science and industrial engineering; New York: Association for Computing Machinery (ACM). (2022), p. 229–236.

[14] Lau R Stevenson F Ong BN Dziedzic K Treweek S Eldridge S . Addressing the evidence-to-practice gap for complex interventions in primary care: a systematic review of reviews protocol. BMJ Open. (2014) 4:e005548. doi: 10.1136/bmjopen-2014-005548, PMID: 24958212 PMC4067819

[15] Ojo SO Olugbara OO Ditsa G. Formal model for e-healthcare readiness assessment in a developing-country context. In: Proceedings of the 4th international conference on innovations in information technology (IIT '07); 2007 Dubai, UAE. p. 41–45.

[16] World Bank. Jordan overview [Internet]. Washington, DC: World Bank; (2025). Available online at: https://www.worldbank.org/en/country/jordan/overview (Accessed April 10, 2025).

[17] World Bank. Data: Jordan [Internet]. Washington, DC: World Bank; (2025). Available online at: https://data.worldbank.org/?locations=JO-XN (Accessed April 10, 2025).

[18] Melhem SJ Nabhani-Gebara S Kayyali R. Leveraging e-health for enhanced cancer-care service models in middle-income contexts: qualitative insights from oncology care providers. Digit Health. (2024) 10:20552076241237668. doi: 10.1177/20552076241237668 38486873 PMC10938624

[19] High Health Council. National health sector strategy 2016–2020 [internet]. Amman: The Hashemite Kingdom of Jordan; (2016). Available online at: https://extranet.who.int/countryplanningcycles/sites/default/files/planning_cycle_repository/jordan/national_strategy_for_health_sector_2016-2020_jordan.pdf (Accessed April 10, 2025).

[20] Electronic Health Solutions. Hakeem Program [Internet] Amman: Electronic Health Solutions. Available online at: https://ehs.com.jo/hakeem-program (Accessed September 4, 2025).

[21] World Health Organization Regional Office for the Eastern Mediterranean. Jordan e-health country profile [internet]. Cairo: WHO EMRO; (2019). Available online at: https://www.emro.who.int/images/stories/ehealth/documents/jordan-ehealth-country-profile.pdf (Accessed September 4, 2025).

[22] Klaib AF Nuser MS. Evaluating EHR and health care in Jordan according to the international health metrics network (HMN) framework and standards: a case study of hakeem. IEEE Access. (2019) 7:83539–48. doi: 10.1109/ACCESS.2019.2924463

[23] Aljassabi A Zieneldien T Kim J Alawneh A Abdel-Razeq H Shamieh O . Cancer care in Jordan: multidisciplinary solutions needed for complex disparities. JCO Glob Oncol. (2025) 11:e2400600. doi: 10.1200/GO-24-00600 40239147 PMC12312410

[24] Gentles SJ Charles C Ploeg J McKibbon KA. Sampling in qualitative research: insights from an overview of the methods literature. Qual Rep. (2015) 20:1772–89. doi: 10.46743/2160-3715/2015.2373

[25] Naderifar M Goli H Ghaljaie F. Snowball sampling: a purposeful method of sampling in qualitative research. Strides Dev Med Educ. (2017) 14:e67670. doi: 10.5812/sdme.67670

[26] Parker C Scott S Geddes A. Snowball sampling. In: SAGE research methods foundations [internet]. London: SAGE; (2019) (Accessed April 10, 2025).

[27] Gagnon MP Ngangue P Payne-Gagnon J Desmartis M. m-health adoption by healthcare professionals: a systematic review. J Am Med Inform Assoc. (2016) 23:212–20. doi: 10.1093/jamia/ocv052, PMID: 26078410 PMC7814918

[28] Akhlaq A McKinstry B Muhammad KB Sheikh A. Barriers and facilitators to health information exchange in low- and middle-income country settings: a systematic review. Health Policy Plan. (2016) 31:1310–25. doi: 10.1093/heapol/czw056, PMID: 27185528

[29] Grol R Wensing M. What drives change? Barriers to and incentives for achieving evidence-based practice. Med J Aust. (2004) 180:S57–60. doi: 10.5694/j.1326-5377.2004.tb05948.x 15012583

[30] Flottorp SA Oxman AD Krause J Musila NR Wensing M Godycki-Cwirko M . A checklist for identifying determinants of practice: a systematic review and synthesis of frameworks and taxonomies. Implement Sci. (2013) 8:35. doi: 10.1186/1748-5908-8-35 23522377 PMC3617095

[31] Gale NK Heath G Cameron E Rashid S Redwood S. Using the framework method for the analysis of qualitative data in multi-disciplinary health research. BMC Med Res Methodol. (2013) 13:117. doi: 10.1186/1471-2288-13-117, PMID: 24047204 PMC3848812

[32] Wiltshire G Ronkainen N. A realist approach to thematic analysis: making sense of qualitative data through experiential, inferential and dispositional themes. J Crit Realism. (2021) 20:159–80. doi: 10.1080/14767430.2021.1894909

[33] Hennink M Kaiser BN. Sample sizes for saturation in qualitative research: a systematic review of empirical tests. Soc Sci Med. (2021) 292:114523. doi: 10.1016/j.socscimed.2021.114523 34785096

[34] Tong A Sainsbury P Craig J. Consolidated criteria for reporting qualitative research (COREQ): a 32-item checklist for interviews and focus groups. Int J Qual Health Care. (2007) 19:349–57. doi: 10.1093/intqhc/mzm042, PMID: 17872937

[35] Fidler CS Kanaan RK Rogerson S. Barriers to e-government implementation in Jordan: the role of Wasta. Int J Technol Hum Interact. (2011) 7:9–20. doi: 10.4018/jthi.2011040102

[36] Cunningham R Sarayrah YK. Wasta: the hidden force in middle eastern society. Westport, CT: Praeger (1993).

[37] Singh A Ravi P. Adoption of e-health platforms by medical practitioners. Health Mark Q. (2022) 39:61–73. doi: 10.1080/07359683.2021.1995637 34720067

[38] De Regge M Decoene E Eeckloo K Van Hecke A. development and evaluation of an integrated digital patient platform during oncology treatment. J Patient Exp. (2020) 7:53–61. doi: 10.1177/2374373518825142 32128372 PMC7036686

[39] Longhini J Rossettini G Palese A. Digital-health competencies among healthcare professionals: systematic review. J Med Internet Res. (2022) 24:e36414. doi: 10.2196/36414, PMID: 35980735 PMC9437781

[40] Von Eschenbach WJ. Transparency and the black box problem: why we do not trust AI. Philos Technol. (2021) 34:1607–22. doi: 10.1007/s13347-021-00477-0

[41] Fink K. Opening the government’s black boxes: freedom of information and algorithmic accountability. Inf Commun Soc. (2018) 21:1453–71. doi: 10.1080/1369118X.2017.1330418

[42] Cui F Zhang X He X Zhao Y Chen L Teng Y . Clinical applications of telemedicine services using a regional telemedicine platform for cancer treatment: a cross-sectional study. BMC Cancer. (2024) 24:808. doi: 10.1186/s12885-024-12563-5 38973010 PMC11229255

[43] Eberts M Capurro D. Impact of electronic health records on the patient–physician relationship. Appl Clin Inform. (2019) 10:729–34. doi: 10.1055/s-0039-1695782 31556076 PMC6760987

[44] Wang S Blazer DG Hoenig H. Can eHealth technology enhance the patient-provider relationship in rehabilitation? Arch Phys Med Rehabil. (2016) 97:1403–6. doi: 10.1016/j.apmr.2016.04.002, PMID: 27109332

[45] Bell SK Mejilla R Anselmo M Darer JD Elmore JG Leveille S . When doctors share visit notes with patients: a study of patient and doctor perceptions of documentation errors, safety opportunities and the patient–doctor relationship. BMJ Qual Saf. (2017) 26:262–70. doi: 10.1136/bmjqs-2015-004697, PMID: 27193032 PMC7255406

[46] Jiang S. How does online patient-provider communication heal? Examining the role of patient satisfaction and communication experience in China. Health Commun. (2019) 34:1637–44. doi: 10.1080/10410236.2018.1517634 30198772

[47] Kooij L Groen WG van Harten WH. The effectiveness of information technology-supported shared care for patients with cancer: a systematic review. Eur J Cancer Care (Engl). (2017) 26:e12497. doi: 10.1111/ecc.12497 28642218 PMC5500776

[48] Ellimoottil C An L Moyer M Sossong S Hollander JE. Challenges and opportunities faced by large health systems implementing telehealth. Health Aff (Millwood). (2018) 37:1955–9. doi: 10.1377/hlthaff.2018.05099, PMID: 30633667

