# Supplementary material for: Scaling holistic e-health solutions in cancer care using a qualitative realist framework
Source: Front Public Health. 2025 Dec 3;13:1617857. doi: 10.3389/fpubh.2025.1617857 (PMC12708916; doi:10.3389/fpubh.2025.1617857)
Supplement: Supplementary file 1 [file Supplementary_file_1.docx]

**Supplementary 1: Participant information Sheet (PIS) for healthcare providers**

**Supplementary 1: Participant Information Sheet (PIS) for Healthcare Providers**

**Study Title: Scaling Holistic E-Health Solutions in Cancer Care Using a Qualitative Realist Framework** [Date: ……/……/2023]

You are being invited to take part in a research study. Before you decide whether or not to take part, it is important for you to understand why the research is being done and what it will involve. Please take time to read the following information sheet carefully before deciding whether or not to participate. If you decide not to take part, there will be no disadvantage to you, and we thank you for your time in considering our project. If, after reading this information sheet, you are still unsure or uncertain about anything, we are happy to answer any questions you may have.

**What is the purpose of the study?**

The primary aim of this study is to explore the experiences, perspectives, and requirements of oncology healthcare professionals regarding the design and implementation of a digital platform for collaborative, patient-centred cancer care. In particular, the study seeks to understand the scalability and implementation challenges and enablers encountered by healthcare providers in delivering holistic supportive care for cancer survivors.

**Why have I been chosen?**

You are invited to participate because you are an oncology healthcare professional with experience in the care of patients with breast and/or colorectal cancer. Your insights are valuable for determining the practical requirements and considerations necessary for implementing a digital health platform in oncology practice.
**There will be no direct or indirect recruitment of patient or caregiver participants as part of this study phase.**

**Do I have to take part?**

No. Participation in this research is entirely voluntary. If you choose to take part after reading this information sheet, we will ask for your informed consent. For face-to-face interviews, written consent will be obtained. For online or telephone interviews, verbal consent will be recorded.
**Verbal consent will be obtained from all participants in online interviews, and written consent will be for any in-person interviews.** You are free to withdraw from this study at any point without disadvantage and without having to provide a reason.

**What will happen to me if I take part?**

You will be invited to participate in a one-to-one interview to share your experiences and opinions as an oncology healthcare professional involved in the care of cancer patients. There will be no requirement to recruit patients or facilitate focus groups for patients or caregivers. The interview will be scheduled at your convenience, either in person or via online platforms.

**What are the possible benefits of taking part?**

Your participation will help us understand the practical and systemic challenges faced by oncology professionals in Jordan, and your input will guide the development of a digital platform intended to support integrated, patient-centred oncology care.

**What are the possible disadvantages and risks of taking part?**

There are no specific risks associated with taking part in this study. However, we understand that your time is valuable and appreciate your willingness to participate.

**What happens when the research study ends?**

You will be under no obligation to volunteer again. Contact details for the principal investigator and project supervisor are included at the end of this information sheet should you wish to discuss the findings.

**Will my taking part be kept confidential?**

All information collected during the course of the study will be kept strictly confidential and securely stored. Only the main researcher (Dr Samar Melhem) and the project supervisors (Prof Reem Kayyali and Prof Shereen Nabhani-Gebara) will have access to this dataset.
Any personal information collected will be immediately destroyed, except as required by University research policy.

**Who is organising and funding the study?**

This study is a collaborative research project between the Pharmacy Department at Kingston University and the Faculty of Pharmacy at the University of Jordan. None of the investigators stands to gain financially from this study.

**What will happen to the results of the research study?**

The results of this research will form part of a joint study with the University of Jordan and will be available at the Learning Resources Centre (library) of the Faculty of Science at Kingston University. In addition, the findings may be presented at national and international conferences and published in scientific journals. You and other individuals will not be identifiable in any such publications, as the results will be reported in aggregate form for the entire group.

**Who has reviewed the study?**

This study has been reviewed and approved by Kingston University Faculty of Science Research Ethics Committee.
The project is coordinated by Dr Samar Melhem , Prof Reem Kayyali and Prof Shereen Nabhani-Gebara.

**Contact for further information**

- **Principal Investigator: Dr Samar Melhem**

**Faculty of Pharmacy. The University of Jordan**

**Queen Rania Street, Amman-Jordan**

**E-mail:** [**sa.melhem@ju.edu.jo**](mailto:sa.melhem@ju.edu.jo)

- **Project supervisor:** Prof. Reem Kayyali
  Faculty of Science, Engineering and Computing, Kingston University London
  Penrhyn Road, Kingston upon Thames, Surrey KT1 2EE
  E-mail: R.Kayyali@kingston.ac.uk
  Tel: +44 (0)20 8417 2561
- **Second supervisor:** Prof. Shereen Nabhani-Gebara
  Faculty of Science, Engineering and Computing, Kingston University London
  Penrhyn Road, Kingston upon Thames, Surrey KT1 2EE
  E-mail: s.nabhani@kingston.ac.uk

If you become concerned about any issue raised by your participation in this study, please contact Dr Melhem or Prof. Kayyali at the details provided above.
