# Supplementary material for: Scaling holistic e-health solutions in cancer care using a qualitative realist framework
Source: Front Public Health. 2025 Dec 3;13:1617857. doi: 10.3389/fpubh.2025.1617857 (PMC12708916; doi:10.3389/fpubh.2025.1617857)
Supplement: Supplementary file 2 [file Supplementary_file_2.docx]

**Interview Guide with Oncology Healthcare Providers**

**Exploring the Use, Recommendation, and Implementation of Digital Health Applications in Supportive Cancer Care**

**Section 1: Personal and Professional Engagement with Digital Health Technologies**

1. Could you describe your personal and professional experiences with mobile health applications or digital tools?
   - What motivates your use (or non-use) of these technologies in your clinical practice?
   - How do you perceive their role in the broader healthcare ecosystem?

1.1. Have you encountered any particular mobile apps, online platforms, or digital resources that you believe are beneficial for cancer patients, particularly in the context of supportive care or self-management?

- What features stood out to you and why?

**Section 2: Current Practices and Attitudes Towards Recommending Digital Resources**

1. What are your current views on recommending or prescribing mobile apps or digital health tools to cancer patients?
   - How do these practices align with your professional values and clinical judgement?

2.1. In your experience, what potential benefits might emerge from recommending mHealth solutions to patients?

- Could you share any examples or scenarios that illustrate these benefits?

2.2. Have you ever recommended digital tools to your patients? If so, what guided your decision-making process?

- What outcomes or feedback have you observed?

**Section 3: Hypothetical Endorsement of Clinically Validated Digital Interventions**

1. If a clinically validated and evidence-based mHealth application were available, would you consider endorsing it in your practice?
   - What conditions would need to be met to justify such endorsement?

3.1. From your perspective, what core features or functionalities should a high-quality digital health app include to support cancer care effectively?

3.2. What type of content (e.g., educational, interactive, monitoring tools) do you believe should be embedded within these tools to maximise patient benefit?

**Section 4: Perceived Benefits of Digital Health Interventions**

1. In your view, what are the broader benefits of health apps for oncology patients and their care trajectories?

4.1. How do you think these digital tools might affect the nature of the patient-provider relationship?

- Could they enhance or diminish relational aspects of care?

4.2. Do you believe that mobile health interventions can strengthen patient loyalty, engagement, and satisfaction?

- What mechanisms might underpin these effects?

4.3. Are there specific patient groups (e.g., by age, diagnosis, digital literacy) that you believe could derive particular benefit from digital health applications?

**Section 5: Barriers and Enablers to Implementation at System and Practice Levels**

1. Based on your clinical experience, what do you perceive as the main barriers to implementing digital solutions in cancer supportive care at scale?

5.1. What are your thoughts on the feasibility and utility of nationwide digital platforms (e.g., patient portals, Personal Health Records) that facilitate communication across the care continuum?

5.2. What structural or contextual factors (e.g., technological infrastructure, funding models, professional culture) might facilitate or hinder the integration of digital health into routine practice?

5.3. Could you elaborate on any patient-level, organisational, or societal determinants (e.g., health literacy, trust, regulation) that might influence the adoption and sustained use of digital health tools?

**Section 6: Workflow Implications**

1. How might the introduction of comprehensive digital health tools impact your day-to-day workflow, time management, or clinical productivity?

6.1. Do you foresee these tools as reducing or increasing your workload? In what ways?

**Section 7: Ethical, Legal, and Data Governance Concerns**

1. What concerns, if any, do you have regarding privacy, security, or legal risks associated with the use of digital health applications?

7.1. How confident are you in current regulatory frameworks and data protection standards to safeguard patient information in this digital context?

**Section 8: Challenges Anticipated for Patient Use**

1. What potential challenges do you think patients might encounter when using mHealth applications, both during treatment and in survivorship?

8.1. How might factors such as age, digital literacy, socioeconomic status, or cultural background influence patients’ ability or willingness to use these technologies?

**Section 9: Integration with Electronic Health Records (EHRs)**

1. What are your views on integrating mHealth applications with existing Electronic Health Records?

9.1. How might granting patients—especially cancer survivors—access to their own EHR data affect care continuity, patient autonomy, or clinical safety?

- Do you have any reservations about such transparency?

**Section 10: Professional Endorsement and Institutional Legitimacy**

1. In your view, how important is it for mHealth solutions to be endorsed by clinical specialists or accredited by authoritative bodies (e.g., Ministry of Health, medical societies)?

10.1. Do you think such endorsements influence patient trust and uptake? Why or why not?

10.2. What further strategies or incentives do you believe could increase healthcare providers’ and patients’ acceptance of digital health solutions in oncology?

**Section 11: Closing Reflections**

1. Is there anything else you would like to add regarding the use, recommendation, or implementation of digital health solutions in cancer supportive care?

11.1. Are there emerging innovations, models, or concerns you believe should be addressed in future research or policy?
