# Supplementary material for: Scaling holistic e-health solutions in cancer care using a qualitative realist framework
Source: Front Public Health. 2025 Dec 3;13:1617857. doi: 10.3389/fpubh.2025.1617857 (PMC12708916; doi:10.3389/fpubh.2025.1617857)
Supplement: Supplementary file 3 [file Supplementary_file_3.docx]

**Supplementary 3: Consolidated Criteria for Reporting Qualitative Research (COREQ) 32-item checklist**

**Supplementary 3: COREQ 32-Item Checklist**

| COREQ Criteria | Criteria Fulfilment in the Current Research |
| --- | --- |
| Domain 1: Research Team and Reflexivity |  |
| Personal Characteristics |  |
| 1. Interviewer/Facilitator | The first author (SJM) performed all the interviews, as noted in the methodology. |
| 2. Credentials | The research team's qualifications were listed in the methods section and their credentials in the title page |
| 3. Occupation | The research team's occupations were provided in the title page |
| 4. Gender | The analysis team comprised four female and two male academics. |
| 5. Experience and Training | The analysis was undertaken by a research team with substantial qualitative research experience in healthcare settings. |
| Relationship with Participants |  |
| 6. Relationship Established | Not stated in the study. No pre-existing relationships existed between research team and participants. |
| 7. Participant Knowledge of the Interviewer | SJM contacted all eligible participants to explain the research objectives and answer any questions. Because the interviewer and oncology healthcare professionals did not know each other, a brief explanation of the research was given at the start of each interview. Only oncology healthcare professionals were recruited, and all interviews were conducted individually. |
| 8. Interviewer Characteristics | The title page and methods section provided qualifications, occupation, gender, and absence of any pre-existing link between interviewer and interviewees. |
| Domain 2: Study Design |  |
| Theoretical Framework |  |
| 9. Methodological Orientation and Theory | Critical realism was the main theoretical orientation; hybrid inductive/deductive thematic framework used for analysis, as noted in the methods. |
| Participant Selection |  |
| 10. Sampling | A purposive sampling of multidisciplinary oncology care providers from multiple health sectors in Jordan was followed by snowball sampling, as detailed in the methods section. Only healthcare professionals were recruited. |
| 11. Method of Approach | SJM approached participants in person or by phone or via WhatsApp for recruitment, as stated in the methodology. |
| 12. Sample Size | 22 interviews with cancer care specialists from different oncology specialties were conducted, stopping at thematic saturation, it is outlined in the methods. |
| 13. Non-Participation | All eligible participants who were approached and agreed to participate as per procedures were enrolled; non-participation was not discussed in the paper. |
| Setting |  |
| 14. Setting of Data Collection | Individual interviews with oncology healthcare professionals were held in a hospital conference room or online (Zoom, Skype, Facetime, Google Meet). No focus groups were conducted. |
| 15. Presence of non-participants | No individuals other than the interview participants were present. All interviews were conducted by the principal investigator, (SJM), as stated in the Methods section. |
| 16. Description of Sample | Table 1 in the results section presents HCP sample characteristics. |
| Data Collection |  |
| 17. Interview Guide | The interview guide is available as Supplementary File 2. |
| 18. Repeat Interviews | No follow-up interviews, as stated in the methods. |
| 19. Audio/Visual Recording | All individual face-to-face or online interviews with HCPs were audio-recorded. |
| 20. Field Notes | Only handwritten notes were taken during individual interviews. |
| 21. Duration | Interview duration specified in the results section. |
| 22. Data Saturation | Sample size determined by data saturation; documented in the methods. |
| 23. Transcripts Returned | Transcripts were not returned to participants, except for two clinical pharmacists who reviewed synthesised data; others were unable due to scheduling. |
| Domain 3: Analysis and Findings |  |
| Data Analysis |  |
| 24. Number of Data Coders | The first author coded all data. Three authors (RK, SN, HM) reviewed and discussed the coding; consensus reached on framework. |
| 25. Description of Coding Tree | The coding structure and framework are described in methods. |
| 26. Derivation of Themes | Themes derived inductively/deductively from data and literature. It is fully described in the methods |
| 27. Software | *NVivo Pro V12* was used to manage and code data. |
| 28. Participant Checking | Synthesized themes were member-checked with an oncology pharmacist and a resident physician to enhance trustworthiness. Reported under 2.4. in methods. |
| Reporting |  |
| 29. Quotations Presented | Participant quotes are included within the Results section; additional illustrative quotations are provided in Supplementary 4. |
| 30. Data and Findings Consistent | All authors validated interpretations, findings, and themes. |
| 31. Clarity of Major Themes | Thematic framework weighted all themes equally. |
| 32. Clarity of Minor Themes | All themes received equal weighting within the framework. |
