# Supplementary material for: Scaling holistic e-health solutions in cancer care using a qualitative realist framework
Source: Front Public Health. 2025 Dec 3;13:1617857. doi: 10.3389/fpubh.2025.1617857 (PMC12708916; doi:10.3389/fpubh.2025.1617857)
Supplement: Supplementary file 4 [file Supplementary_file_4.docx]

**Supplementary 4. Additional quotations by second-order code¹**

| Deductive subtheme | Barrier / Facilitator | Quotation (verbatim) | Participant / Role |
| --- | --- | --- | --- |
| A. Innovation | Barrier | “I’m not sure if the apps can actually improve the patients’ outcomes, but I believe that educational, e-services, and communications would be the primary benefit.” | HCP8, surgical oncologist, KHCC, semi-governmental, male, 52 years |
|  | Barrier | “While I am familiar with the concept of health apps, which can be found in the app store, I am not aware of any that facilitate communication between doctors and their patients.” | HCP12, oncology pharmacist, private sector, female, 36 years |
|  | Barrier | “I don’t think it’s bandwidth, but we need new handheld devices and enough IT people to maintain the service.” | Clinical nurse specialist, KHCC, semi-governmental, female, 39 years |
|  | Barrier | “The platform should have an intuitive and easy-to-use interface that accommodates different user needs, such as voice dictation and specialised functions for pharmacists and doctors. Before using the platform, it needs to be tested. As a PharmD, I expect the platform to support my work, but doctors may need different functionalities. So, ultimately, a user-friendly technology that streamlines healthcare professionals’ workflows would improve patient care.” | HCP11, oncology pharmacist, private sector, female, 37 years |
|  | Barrier | “It should be easily accessed on mobile phones, or desktops.” | HCP15, clinical nurse specialist, KHCC, semi-governmental, female, 37 years |
|  | Facilitator | “Yes, we need prominent cancer doctors to support the app, and it should have transparent affiliations with institutions or the Ministry of Health to increase user adoption.” | HCP12, oncology pharmacist, private sector, female, 36 years |
|  | Facilitator | “Most of them [patients] have insurance, so I doubt they will pay for such a service, especially when you start it…” | HCP7, medical oncologist, public sector, male, 49 years |
|  | Facilitator | “The system should be open-source for interoperability with other providers, while remaining proprietary to guarantee that only eligible patients access our services.” | HCP2, senior oncology manager, KHCC, semi-governmental, female, 56 years |
|  | Facilitator | “Initially, virtual clinic access should be restricted to a specific patient group, allowing for evaluation of interest from both patients and providers prior to expanding the programme.” | HCP1, surgical oncologist, university hospital director, semi-governmental, male, 69 years |
| B. Patient | Barrier | “Maybe patients with more curable cancers benefit more and are driven to use the app to improve quality of life… people with advanced tumours, I am not sure if they will use it; it depends on patient preferences, and we need to take each case by case.” | HCP15, clinical nurse specialist, KHCC, semi-governmental, female, 37 years |
|  | Barrier | “Cancer patients have psychological concerns such as anxiety and despair, so I am not sure whether they will use the app for self-management or lifestyle changes if they lack motivation. I think we need to examine what the patient needs and think carefully about the app’s use. Psychological support is vital, and many patients require it, so teleconsultations can be beneficial. You should give it some thought.” | Clinical nurse specialist, KHCC, semi-governmental, male, 39 years |
|  | Barrier | “Patients should be compliant and record their symptoms and how they feel, but they become bored and, as you know, even with medicines we have non-adherence.” | HCP11, oncology pharmacist, private sector, female, 37 years |
|  | Barrier | “…I envisage most likely those well-educated, tech-savvy, predominantly good-prognosis patients…” | HCP10, medical oncologist, private sector, female, 57 years |
|  | Barrier | “…Also, we must consider the patient’s mental, cognitive, and age-related factors, as well as whether they have anxiety, and you must tailor the app content to several variables…” | HCP9, surgical oncologist, military services, male, 63 years |
|  | Facilitator | “If patients believe their clinicians are more accessible because of these tools, I believe satisfaction with care and the quality of healthcare as a whole will improve.” | Senior clinical fellow, KHCC, semi-governmental, male, 40 years |
|  | Facilitator | “If the app is useful to its users, such as by providing them with specialised information on their condition, it will serve as a strong incentive for others to download it and engage in the community.” | HCP12, oncology pharmacist, private sector, female, 36 years |
|  | Facilitator | “Patients with cognitive decline are common, and when I explain it to them, they ask me again when they get home because they’ve forgotten. This app could be used as a backup to document and distribute information to these patients.” | HCP3, medical oncologist, private sector, female, 57 years |
|  | Facilitator | “Patients with cognitive impairment or the elderly commonly call me when they get home to enquire about their treatment and what to do. During appointments, some patients may not understand everything.” | HCP4, haematologist-oncologist, military services, male, 47 years |
|  | Facilitator | “The platform should have an intuitive and easy-to-use interface… ultimately, a user-friendly technology that streamlines healthcare professionals’ workflows would improve patient care.” | HCP11, oncology pharmacist, private sector, female, 37 years |
|  | Facilitator | “As a doctor, I don’t see much point in supportive cancer apps. They might be good for patient education and supportive care, but I’d rather start and focus on telemedicine programme development and implementation.” | HCP1, surgical oncologist, university hospital director, semi-governmental, male, 69 years |
| C. Healthcare Professionals | Barrier | “Granting patients access to clinical notes is problematic, as these notes are intended for professional use and may not be suitable for patient reading. Simplifying these notes for patients would be beneficial, but adding this task to healthcare professionals’ workload is impractical. Therefore, while patient portals should enhance existing e-services, the responsibility for managing this remains unclear.” | HCP10, senior fellow oncologist, KHCC, semi-governmental, male, 40 years |
|  | Barrier | “…Initially, implementing our electronic medical records system required archiving paper files and manual data entry, a time-consuming process. For medical histories over five years, paper files are still necessary. Given the workload, portals seem unnecessary and burdensome. Thus, dedicated service providers should develop these portals.” | HCP20, fellow oncologist, university hospital, semi-governmental, female, 30 years |
|  | Barrier | “Hospitals face a critical shortage of skilled clinical nurse specialists, limiting care to inpatients and failing to meet the informational needs of patients and caregivers. Enhancing nurse training and system use is essential for delivering greater value.” | HCP15, clinical nurse specialist, KHCC, semi-governmental, female, 37 years |
|  | Barrier | “Some doctors are resistant to patients consulting external sources, feeling it undermines their authority and disrupts their work.” | HCP11, oncology pharmacist, private sector, female, 37 years |
|  | Barrier | “Establishing clear remuneration models for teleconsultations and virtual care was cited as essential to incentivise healthcare professional participation and sustain digital service delivery.” | HCP13, oncology pharmacist, private sector, female, 33 years |
|  | Facilitator | “To carry out this programme, you should establish digital care teams.” | HCP2, senior oncology manager, KHCC, semi-governmental, female, 56 years |
|  | Facilitator | “The software should be developed to add synergy to my work and help me undertake my responsibilities more efficiently; therefore, if it can help me complete certain tasks electronically and provide supportive care services remotely, that would be acceptable.” | HCP22, fellow oncologist, university hospital, semi-governmental, male, 31 years |
|  | Facilitator | “Educational apps or appointment scheduling tools will not harm provider–patient relationships; they could enhance communication if they offer robust educational support. We often need to correct misinformation that patients gather from various sources, which can unnecessarily increase their anxiety.” | HCP2, senior oncology manager, KHCC, semi-governmental, female, 56 years |
|  | Facilitator | “Constraints in time, finances, and standardised materials hinder comprehensive patient education. A national platform for patient-focused content would help, but personalised treatment plans still require specialised staff. While machine learning could theoretically create personalised plans, it is uncertain when this will be applied in clinical practice.” | HCP20, fellow, university hospital, semi-governmental, female, 30 years |
|  | Facilitator | “We really do not have the time, and we work under significant pressure, so either this needs to be part of our jobs or we need to receive additional pay. For example, as a clinical nurse, they would allocate me 20 hours per week of digital support work, which might be overtime or part of my regular working hours. I believe it is unrealistic and impractical to include health practitioners in the app without compensating them and freeing up their time.” | HCP15, clinical nurse specialist, KHCC, semi-governmental, female, 37 years |
| D. Organisational Context | Barrier | “For the platform’s success, leadership buy-in and top-down implementation are vital, as are training and reimbursement for health teams to ensure they can dedicate sufficient time to app usage.” | HCP15, clinical nurse specialist, KHCC, semi-governmental, female, 37 years |
|  | Barrier | “We have a portfolio of projects to evaluate, so we must also consider how each project, individually, can contribute to resolving problems in service delivery in terms of cost-effectiveness and impact on performance.” | Senior oncology manager, KHCC, semi-governmental, female, 56 years |
|  | Barrier | “Creating a digital platform and integrating it with hospital records can seem frivolous against the backdrop of current care models and limited resources, leading decision-makers to balk at reallocating funds from more critical expenditures.” | HCP1, surgical oncologist, university hospital director, semi-governmental, male, 69 years |
|  | Barrier | “The hierarchical structure of our organisation complicates innovation adoption because of formalities. Solutions need to be enterprise-focused and cost-effective to attract management support.” | HCP8, surgical oncologist, KHCC, semi-governmental, male, 52 years |
|  | Barrier | “The devil is in the details, so if we are talking about a comprehensive digital platform for cancer patients, that is a major project.” | HCP14, clinical nurse specialist, KHCC, semi-governmental, female, 34 years |
|  | Barrier | “If change is implemented in a top-down manner, the status quo will likely return once a top-level manager leaves the organisation.” | HCP8, surgical oncologist, KHCC, semi-governmental, male, 52 years |
|  | Facilitator | “To reduce workflow disruption, I suggest creating a dedicated department for these services, staffed by trained personnel responsible for devising educational programmes and supportive interventions for patients and healthcare professionals.” | HCP7, medical oncologist, public sector, male, 49 years |
|  | Facilitator | “Collaboration is essential to develop a national patient access system that securely connects our facility with most others, preventing a scenario where each hospital operates its own isolated portal or app.” | HCP1, surgical oncologist, university hospital director, semi-governmental, male, 69 years |
|  | Facilitator | “If the platform is not effective or does not enhance services, users will find alternative channels, merely changing our approach. Success hinges on reducing costs and elevating service quality.” | HCP9, oncology radiologist, public sector, female, 46 years |
| E. Social Context | Barrier | “The impact of the app on patient relationships is uncertain due to cultural norms valuing non-professional medical advice. People often choose doctors based on personal recommendations or social media, rather than online searches, especially for serious conditions. In cancer care, choices are limited by the few available specialists, treatment centres, and the high cost of care.” | HCP20, fellow, university hospital, semi-governmental, female, 30 years |
|  | Barrier | “People resort to ‘Wasta’ to access and maximise services. However, if the system were fair, efficient, and reliable, the need for Wasta would be moot.” | HCP1, surgical oncologist, university hospital director, semi-governmental, male, 69 years |
|  | Barrier | “Telemedicine has historically benefited underserved communities, yet assessing their digital healthcare readiness is essential.” | HCP15, clinical nurse specialist, KHCC, semi-governmental, female, 37 years |
|  | Facilitator | “With effective promotion across multiple levels, especially in large cities where internet and smartphone access is widespread, the use of these tools is likely to increase significantly.” | HCP21, fellow, university hospital, semi-governmental, male, 31 years |
|  | Facilitator | “Many people may be resistant to the idea of virtual doctor visits because they lack familiarity with the technology involved. It will take some time, and it will be especially difficult to convey the message to patients in disadvantaged settings such as refugee camps.” | HCP15, clinical nurse specialist, KHCC, semi-governmental, female, 37 years |
| F. Economic and Political | Barrier | “There is a worry that unchecked, profit-motivated development of digital health apps could lead to more chaos and disruption in healthcare. This includes concerns over doctors prioritising profit through e-consultations.” | HCP3, medical oncologist, private sector, female, 57 years |
|  | Barrier | “Regulatory processes slow us down. For digital initiatives to be sustainable, it is crucial to involve multiple sectors, including the Telecom Ministry, Ministry of Health, and medical associations, to develop frameworks.” | HCP1, surgical oncologist, university hospital director, semi-governmental, male, 69 years |
|  | Barrier | “At present, there is no overarching national digital health strategy to direct the healthcare sector’s transformation.” | HCP1, surgical oncologist, university hospital director, semi-governmental, male, 69 years |
|  | Barrier | “Government subsidies are not available for the project, requiring buy-in from the payer’s system. If a private provider is involved, they will seek profits. Patients are unlikely to pay for this service at the outset, so covering development costs is possible, but we need to consider other expenses.” | HCP1, surgical oncologist, university hospital director, semi-governmental, male, 69 years |
|  | Facilitator | “As technology becomes more affordable, it offers potential improvements. However, success requires collective effort. The main challenge lies in planning, execution, and financing. Without addressing these, progress will remain stalled.” | HCP4, haematologist-oncologist, military services, male, 47 years |
|  | Facilitator | “Our telemedicine initiatives in Jordan and for crisis management in neighbouring countries (Lebanon) were focused but limited, scattered, and unsustainable. KHCC has a programme for international collaboration with specialists.” | HCP4, haematologist-oncologist, military services, male, 47 years |
|  | Facilitator | “Telemedicine is essential in times of crisis; the armed forces make use of it on an as-needed basis.” | HCP6, surgical oncologist, military services, male, 63 years |

**¹ Footnote :***Each second-order code is evidenced by at least one verbatim quotation either in the main Results text or in this Supplementary Table. Where an additional quotation would be redundant, the code is evidenced in the main text. Where a single quotation supports multiple codes, this constitutes shared evidence.*
